# Al Alloy Melting Behavior and Interfacial Reactions with Steel Under Natural Convection

**DOI:** 10.3390/ma17215140

**Published:** 2024-10-22

**Authors:** Shen-yang Song, Jing Li

**Affiliations:** State Key Laboratory of Advanced Metallurgy, University of Science and Technology Beijing, Beijing 100083, China; lijing@ustb.edu.cn

**Keywords:** mass transfer, Al melting, natural convection, steelmaking process, inclusion

## Abstract

The Al alloy melting behavior and interfacial reactions during the steelmaking process of high-Al automotive steel were investigated in this study. The total dissolution time of Al bars (20 × 20 × 80 mm) in molten steel was quite short, decreasing from 21.4 s to 10.0 s with an increase in bath temperature from 1580 to 1620 °C. The Al alloy melting process at the molten steel temperature of 1600 °C included the formation of a solidified steel layer, the latter’s rapid melting, and Al alloy normal melting, while at 1600 °C, the process included a second increase in the thickness of the solidified layer. Because steel elements such as [Fe], [C], [O], and [N] could diffuse during the whole Al alloy melting process, an Fe (Al)-FeAl-FeAl_2_-Fe_2_Al_5_-Al diffusion layer along the direction of the Al-rich matrix could be found at the Fe–Al interface. Moreover, Fe_x_O, Al_2_O_3_, and unstable AlN inclusions could be observed in the FeAl layer. This study also investigated how to reduce the number of these easily formed inclusions. Decreasing the pre-heating process time and dissolved oxygen content could be useful in decreasing Fe_x_O and Al_2_O_3_ inclusion formation. Some small-sized AlN inclusions formed in the center of the Al bars where they could not come into contact with the molten steel directly during the melting process; even for the immersion time of only 1 second, these inclusions were not stable in molten steel at the refining temperature and disappeared during the melting process.

## 1. Introduction

The balance between high strength and light weight is a key parameter for automotive steel, to which aluminum is usually added to reduce steel density [1]. Currently, the development of advanced high-strength steel that contains manganese and varied aluminum amounts is of significant interest for use in steel plants [2,3].

Steel mills still face problems in stably producing advanced high-strength steel. Many kinds of Al alloys are added to liquid steel during the basic oxygen furnace (BOF) tapping and secondary refining processes, with the addition of Al alloys causing significant decreases in molten liquid temperature. However, there is little research on the initial melting behavior and interactions of Al alloys. Most steel researchers have studied the solid–liquid reaction under natural convection, focusing on the scrap melting process. Some studies focused on influencing factors such as scrap size, pre-heating temperature, carbon content, and bath temperature, and considered regular-shape scrap bars as experimental materials [4,5]; they showed that the scrap melting process is a dissolution phenomenon involving both the heat and mass transfers between the liquid–solid phases and that the melting rate is dependent on the mass transfer. These experiments presented room for improvement, for example, in the pre-heating of the scrap bars, which were hung above the surface of the hot metal until their temperature approached the bath temperature; hence, the formation and re-melting of a solidified steel layer that affected the following heat and mass transfers were ignored [6,7]. Gao provided an outline of the whole scrap melting process [8]: Firstly, a solidified steel layer appears. Secondly, the latter melts again until it completely disappears. Thirdly, the scrap metal starts to melt, accompanied by heat and mass transfers. Finally, the scrap bar completely dissolves into the molten steel.

While many results on the scrap melting process have been applied to the prediction of Al melting in molten steel, some problems still require attention. The melting point of aluminum is 660 °C and the bath temperature is usually above 1600 °C [9,10]. Due to the large temperature difference, the heat transfer between liquid Fe and solid Al causes the latter to partly melt when the steel shell still exists. In this situation, the solid shell continues to grow to maximum thickness depending on the inner heat transfer balance of the Al bar [9].

At present, there is a lack of studies focusing on mass transfer in the melting process of aluminum. Furthermore, few researchers have studied inclusion formation in the initial melting stage. Although the thermodynamic formation of deoxidation products (Al_2_O_3_ inclusions) and the kinetic removal process of Al_2_O_3_ inclusions have been studied widely in regular low-Al killed steel, the inclusion formation mechanism in steel containing high Al content requires further investigation [11,12,13]. Van Ende [11] found that the Al_2_O_3_ inclusion phenomenon takes place in the early stage of Al deoxidation. The conclusion was that the number of Al_2_O_3_ inclusions increases steeply with the increase in dissolved oxygen content in molten steel. However, their research study did not consider whether the inclusion species could change with different contact times.

In this study, Al alloy melting experiments under natural convection were carried out in the laboratory. Rod-shaped aluminum was placed into contact with molten steel at 1550–1620 °C for a short time. SEM-EDS observations and thermodynamic calculations were used to clarify the process of mass transfer, the reaction, and inclusion formation in the initial stage of aluminum alloy melting. Our ultimate goal was to provide a theoretical basis for increasing aluminum content in steel.

## 2. Experiments

**Figure 1:**
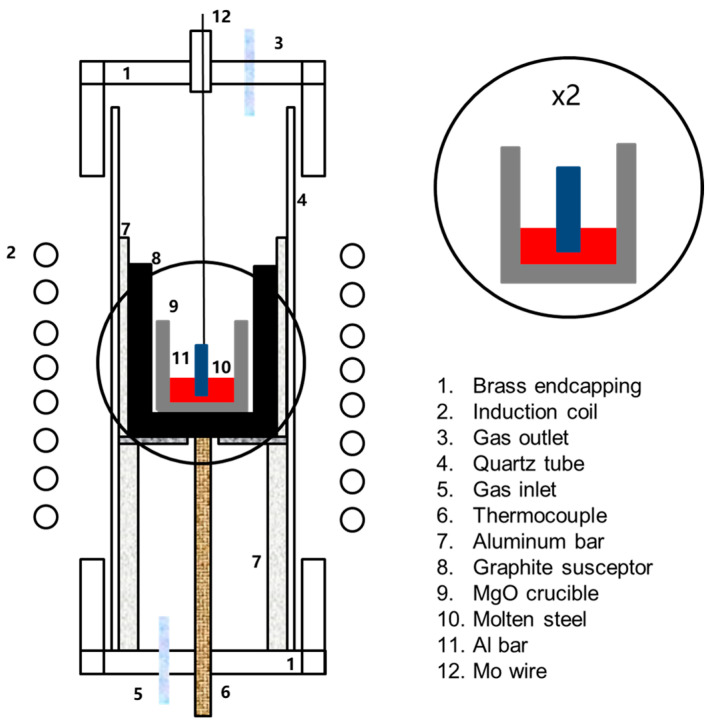
Schematic of experimental apparatus.

A total of 500 g of steel was placed in a MgO crucible (ID: 62 mm; OD: 71 mm; H: 80 mm), and some Fe_2_O_3_ powder was also put into the crucible in order to adjust the dissolved [O] in steel. The MgO crucible was placed in a protective graphite crucible before heating it in a tube furnace, which was equipped with a B-type thermocouple and controlled with a PID (proportional–integral–derivative) controller. The furnace was heated at a rate of 10 °C/min until 1400 °C and at 5 °C/min thereafter. Schematic of experimental apparatus was shown in Figure 1. We started injecting high-purity argon (Ar ≥ 99.99 mass%) into the furnace when the temperature was 600 °C at the gas flow rate of 1.5 L/min, adjusting the latter to 2.5 L/min when the furnace temperature was above 1500 °C.

The molten steel was heated until it reached the target temperature. Then, Al bars were immersed in it by using a Mo wire, the other side of which was tied to a uniform speed motor, which ensured that the falling speed was consistent. The Al bars were not pre-heated to prevent them from melting before the experiments. In this study, as there were few inclusions in the molten steel, the dissolved oxygen was considered to be the total oxygen, which was analyzed with an oxygen/nitrogen/hydrogen analyzer (EMGA-830; HORIBA, Kyoto, Japan).

The Al bars were cut in a cubic shape with a wire cutting machine and were then ground and polished; their final dimensions were 20 × 20 × 80 mm. Each Al bar was immersed in molten steel as quickly as possible, and more than 40 mm of the bar was kept under the surface. Then, the metal bar was quickly taken out and put into an ice–water mixture for rapid cooling. The contact time of the bars in liquid steel varied from 1 to 20 s. The composition of the two materials is shown in Table 1. The experimental temperatures were chosen as 1580 °C, 1600 °C, and 1620 °C because the process in question occurs during the tapping and secondary refining processes at 1580–1620 °C. Each experiment was repeated three times at the same temperature.

After the reaction, a scanning electron microscope (SEM; FEI Quanta-250; FEI Corporation, Hillsboro, OR, USA) was used to observe the surface morphology of the samples, and an energy-dispersive spectrometer (EDS; XFlash 5030; Bruker, Berlin, Germany) was used to analyze the reaction interface composition and the longitudinal section of the Al bars.

## 3. Results and Discussion

### 3.1. Heat Transfer Mechanism of Al Alloy during Melting Process

#### 3.1.1. Morphological Change in Al Bars

**Figure 2:**
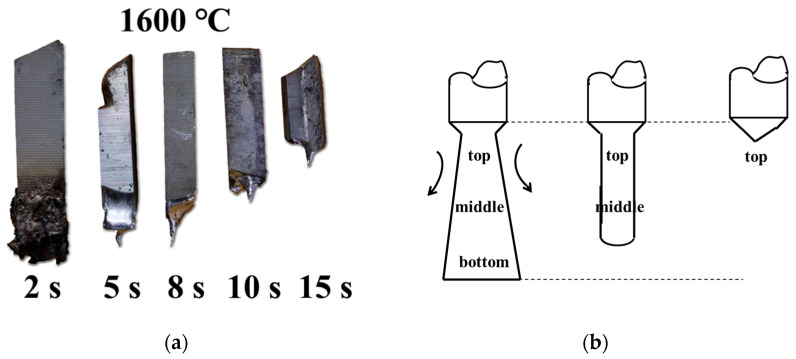
Typical morphological change in Al bar after melting process at 1600 °C: (**a**) actual morphological change and (**b**) schematic diagram of melting process under natural convection.

The morphological change in the Al bars during the melting process is discussed first. Figure 2 shows the typical appearance change in an Al bar at 1600 °C. Molten steel solidified on the Al bar in the initial stage when the bar was immersed in it. It can be seen that the diameter and length of the Al bar decreased during the process, and its shape changed from cubical to conical with the increase in melting time. The experimental results indicate that the heat and mass transfers occurred in both the radial and axial directions of the Al bar.

The typical morphology of the Al bars after the reaction was the conical shape caused by the natural convection of molten liquid steel, as shown in Figure 2, where the radial diameter at the top was larger than that at the bottom. The density of liquid steel increases at low temperature. Thus, given the difference in liquid steel density along the vertical solid–liquid surface because of the heat transfer between the two materials, the molten medium started to flow in the direction of the arrow shown in Figure 2b; high-density liquid steel was constantly replaced by low-density molten steel, ultimately inducing natural convection [8].

**Figure 3:**
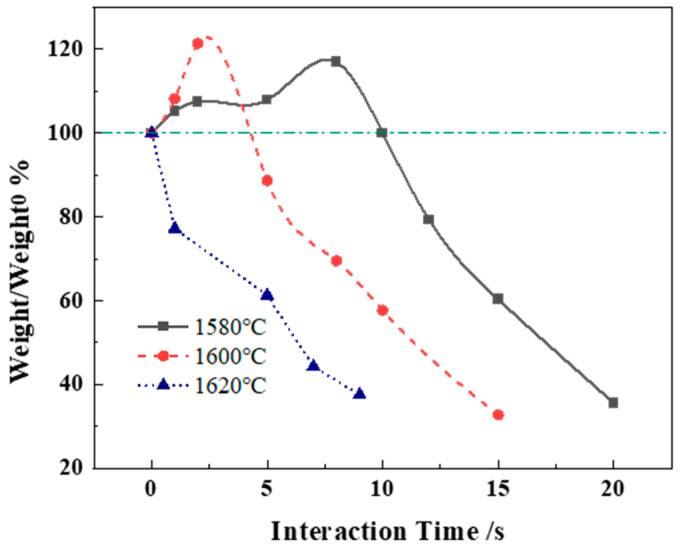
Changes in m/m_0_ of Al bar during melting process.

The whole Al alloy melting process can be described in three steps: the formation of a solidified layer, the latter’s rapid melting, and Al alloy normal melting. In the first step, hot steel cools on the surface of the Al bar, and a solidified layer forms quickly, with its thickness being dependent on the heat transfer. In the second step, the thickness of the formed steel layer decreases until the latter disappears completely, and the surface of the Al bar is again exposed to molten steel. In the end, the Al bar starts melting at a stable dissolution speed which is dependent on the heat balance between the two materials. Figure 3 shows the weight change curves of the aluminum bars at 1580 °C, 1600 °C, and 1620 °C, and the green line in the figure was the 100% standard line.

We recorded an increase in dissolution time, as shown in Figure 3, which was attributed to the bath temperature decrease. The results of the dissolution time were obtained by taking the average value of three repeated experiments at the same temperature. In the test at 1620 °C, the total dissolution time was 10 s, and the solidified layer was not observed. The weight of the Al bars increased in the first 5 s at 1600 °C, indicating the presence of a steel layer at that time. The dissolution time was 14.7 s at 1600 °C. The Al bars dissolved in molten steel at 1580 °C needed a longer time compared with the observations at higher temperatures (21.4 s). The solidified layer had a second increase in thickness at 1580 °C, which was not found in the other high-temperature tests. The length of the Al bars was 80 mm, of which about 20 mm, for ensuring the safety of the experiments, was left above the surface, while the rest was immersed in steel. The weight of the non-immersed part was about 30% of the total Al bar. It could be seen that at the end point of the experiments, there was still a 30% proportion of the whole weight. Pehlke found that the thickness of the steel layer has a downward trend because of the bath temperature increase. The weight of the steel layer decreased when the temperature increased in this study, which is in good agreement with the research results obtained by Pehlke [6].

#### 3.1.2. Heat Transfer Mechanism in Different Aluminum Alloy Melting Processes

The dissolution of the Al bars in molten steel was affected by both heat and mass transfers. The former is analyzed in this section.

The three-dimensional unsteady thermal conductivity differential equation of an Al bar in the radial direction is written as follows [9]:

(1) $$\rho \cdot c_{p}\cdot \frac{\partial T}{\partial t}=\frac{\partial }{\partial r}\cdot (\lambda \cdot r^{2}\cdot \frac{\partial T}{\partial r})+S$$

(2) $$\lambda =a\cdot \rho \cdot c_{p}$$

In the equation, *a* is the thermal diffusion coefficient (m^2^/s). *c_p_* is the specific heat capacity (j/ (kg·k)). *ρ* is the density of aluminum (kg/m^3^). λ is the heat conductivity coefficient (W/(m·k)). *S* is the heat source (W/m^3^).

The latent heat released or absorbed during the phase transition at the solid Al–liquid steel interface, where the mass transfer moving front can be considered the heating boundary, should be considered carefully. The temperature compensation method was applied to calculate the latent heat in the other areas of the Al bar. Equation (1) was then rewritten as Equation (3)

(3) $$\frac{\partial T}{\partial t}=a\cdot \left(\frac{\partial^{2}T}{\partial r^{2}}+\frac{2}{r}\cdot \frac{\partial T}{\partial r}\right)$$

where t_clear_ is the assumed solidified layer’s total dissolution time point (s). R_0_ is the initial radius of the Al bar (m). T _inner-Al_ and T _inner-steel_ are the temperatures of the Al–Fe interface close to the Al and Fe sides (°C), respectively. T_m1_ and T_m2_ are the steel and Al melting points, 1421 °C and 660 °C, respectively. T_bath_ is the bath temperature (°C). The latent heat of Al was calculated to be 387.8 kJ/kg and that of steel 270 kJ/kg [9].

The heat transfer control equation is shown in Equation (4) for the solidified steel side when t < t_clear_

(4) $$\frac{\partial T}{\partial t}=a_{steel}\cdot \left(\frac{\partial^{2}T}{\partial r^{2}}+\frac{2}{r}\cdot \frac{\partial T}{\partial r}\right)\;\;\;\;R_{0}\leq r\leq R_{t}$$

where *a_steel_* is the thermal diffusion coefficient of steel (m^2^/s).

For the Al side of the contact surface, the equation of heat transfer dependency can be written as follows:

(5) $$\frac{\partial T}{\partial t}=a_{Al}\cdot \left(\frac{\partial^{2}T}{\partial r^{2}}+\frac{2}{r}\cdot \frac{\partial T}{\partial r}\right)\;\;\;\;0\leq r\leq R_{0}$$

where *a_Al_* is the thermal diffusion coefficient of Al (m^2^/s).

When t > t_clear_, the equation of heat transfer dependency of the whole melting process is the same as Equation (5).

#### 3.1.3. Melting Boundary Condition of Al Bars

In the melting experiments, the Al bars had a boundary condition, due to their central symmetry, as shown in Equation (6).


(6) $$r=0,\;\frac{\partial T}{\partial r}=0$$

The solidified steel layer does not fit tightly on the Al bar surface well, so the thermal resistance (R) interface should be considered at r = R_0_. R _interface_ disappears when the solidified steel layer or the Al bar dissolves in steel completely. The value of the R interface was 3.0 × 10^−4^ m^2^·k/W in this study, in agreement with the range from 1.9 × 10^−4^ m^2^·k/W to 9.1 × 10^−4^ m^2^·k/W in previous research [14,15]. It was assumed that t melt was the starting time point of the Al bar; hence, the heat boundary condition under the different melting conditions could be considered as discussed below.

When the thickness of the solidified steel layer increases,

(7) $$\lambda \cdot {\frac{\partial T}{\partial r}|}_{r=R}=\rho \cdot L_{m}\cdot \frac{dR}{dt}+h\left(T_{bath}-T_{m1}\right)$$

t < t _melt_ < t_clear_, R _interface_ = 3.0 × 10^−4^ m^2^·k/W,

(8) $$\lambda_{Al}\cdot \frac{\partial T}{\partial r}=\lambda_{steel}\cdot \frac{\partial T}{\partial r}=\frac{T_{inner-steel}-T_{inner-Al}}{R_{T}}$$

t _melt_ < t < t_clear_, R _interface_ = 0, T _inner-steel_ = T _inner-Al_,

t ≥ t _melt_ > t_clear_, R _interface_ = 0, T _inner-steel_ = T _inner-Al_,

(9) $$\lambda \cdot {\frac{\partial T}{\partial r}|}_{r=R}=\rho \cdot L_{m}\cdot \frac{dR}{dt}+h\left(T_{bath}-T_{mi}\right)$$

(10) Nu=2+0.6Re0.5·Pr1/3

(11) $$h=\frac{\lambda_{f}\cdot Nu}{d_{p}}$$

(12) Pr=ν/a

where *L_m_* is the latent heat (j/kg), *dR*/*dt* is the moving speed of the melting boundary, *Nu* is the Nusselt number, Pr is the Prandtl number, λ steel is the thermal conductivity of liquid steel (W/(m·k)), and ν is the kinematic viscosity of liquid steel (m^2^/s).

**Figure 4:**
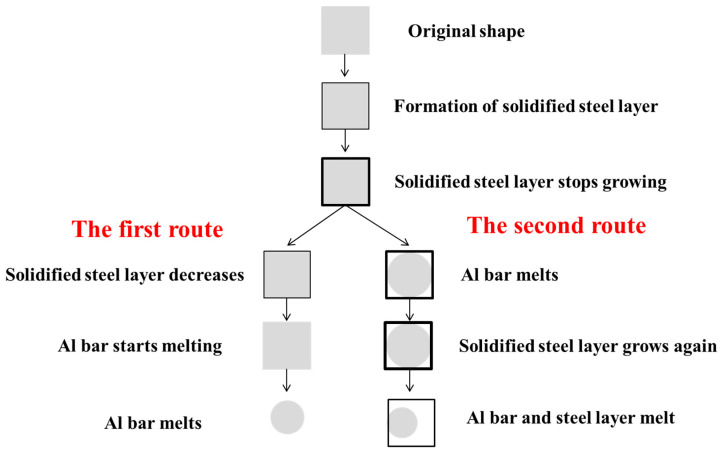
Routes of melting process of Al rod in liquid steel.

The two possible routes of the melting process of an Al rod in liquid steel are shown in Figure 4.

The heat transfer from molten steel to the Al bar at the interaction surface starts when the latter is immersed in the former. At the beginning, the value of the heat flux inside the Al bar is higher than that at the solid–liquid surface. Energy is needed to balance the unbalanced heat flux at the interaction surface, so molten steel cools to release latent heat. As a result, a solidified steel layer forms around the Al bar.

Then, high-temperature molten steel flows from farther away, increasing the heat around the Al bar. Hence, the solidified steel layer stops growing. In the next stage, the heat flux demand inside the Al bar is smaller because of the temperature increase in the whole Al bar, so the solidified steel layer quickly dissolves into molten steel. This melting process is similar to that of scrap steel dissolution [16,17].

In our experiments, we observed another melting process of the Al bars in liquid steel, whereby the Al rods melted before the solidified steel layer could, inducing a second increase in the latter’s thickness.

In this case, the first stage is the same as above. However, in the second stage, the R interface disappears because part of the Al bar liquefies, which is attributed to the low melting point of aluminum. This promotes a drastic increase in the heat flux, the value of which inside the Al bar is again higher than that at the solid–liquid surface; therefore, molten steel has to release more energy to balance the heat transfer. As a result, the thickness of the steel layer increases again. Finally, the heat consumed inside the Al bar is smaller than the inlet heat from molten steel; hence, the Al bar and the solidified steel layer dissolve at the same time.

### 3.2. Mass Transfer Mechanism of Al Alloy during Melting Process

**Figure 5:**
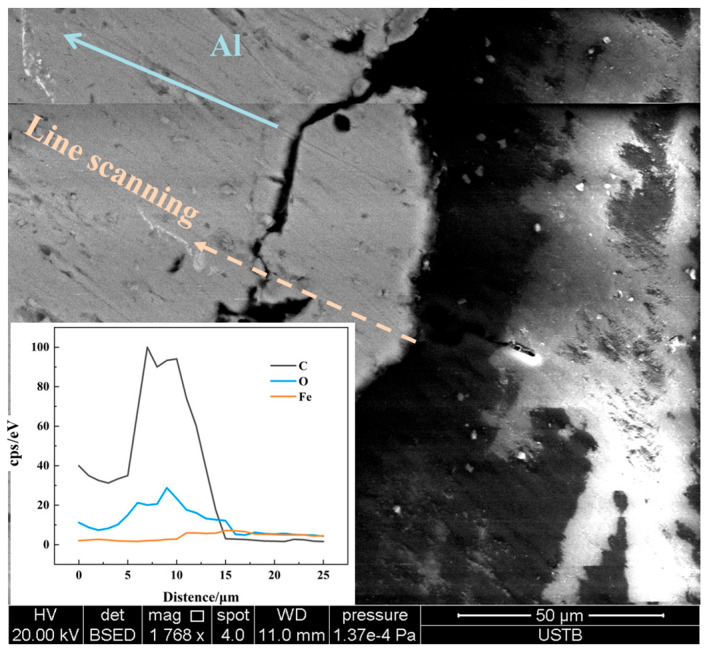
Line-scanning result of composition of Al bar cross-section.

Figure 5 shows the line-scanning result of an Al bar’s cross-section at 1600 °C for 5 s, where a carbon-containing layer whose thickness was about 10 μm was discovered. The carbon diffused from the molten steel pool to the Al bar surface because of the carbon concentration gradient, even though only 0.129% [C] was contained in the molten steel in this study. Specifically, the carbon concentration gradient induces the diffusion of this element from molten steel to the Al bar to decrease the melting point of the latter. This phenomenon is in line with research results on scrap steel melting in hot metal with a larger carbon concentration gradient [18,19,20].

**Figure 6:**
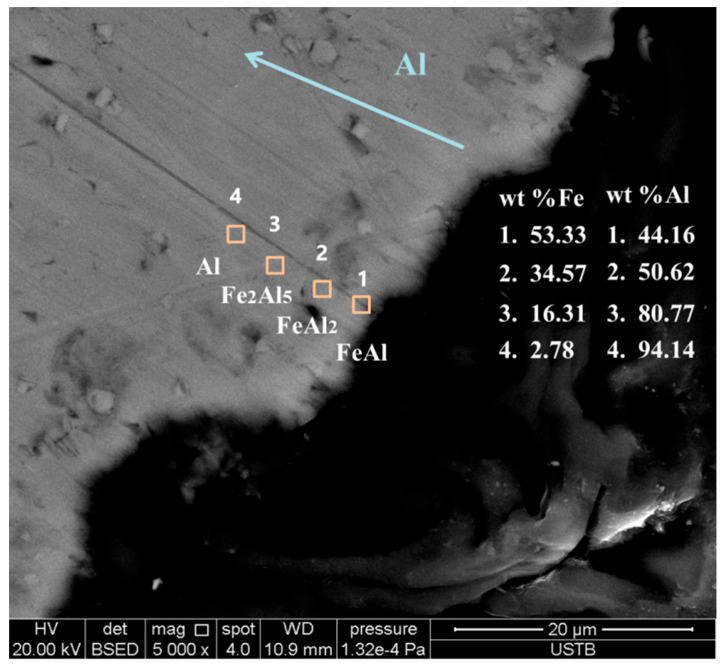
Different diffusion layer areas in Al bar cross-section.

The SEM-EDS dot scan mode was used in order to distinguish the different diffusion layers, and every dot was tested three times to obtain the average composition. In the SEM-EDS image in Figure 6, it can be seen that a successive FeAl layer was found close to the Al–Fe surface; further, FeAl_2_ and Fe_2_Al_5_ layers were found close to the Al zone and in the Al-rich zone direction, respectively, with an average composition of 16 to 30 wt% Al for the former and 50.62 wt% Al and 34.57 wt% Fe for the latter.

Based on the composition analysis of the diffusion layer areas shown in Figure 6, it can be seen that the diffusion layer was composed of Fe(Al)-FeAl-FeAl_2_-Fe_2_Al_5_-Al along the direction of the Al-rich matrix. The Fe_2_Al_3_ layer from the previous investigation by the present authors was not observed here [21]. The reason is that in the previous study, the interaction area between liquid Fe and Al was small, as a quartz tube was used to bring only a small volume of molten Fe into contact with the Al. In addition, more contact time would have allowed more second phases to grow.

**Figure 7:**
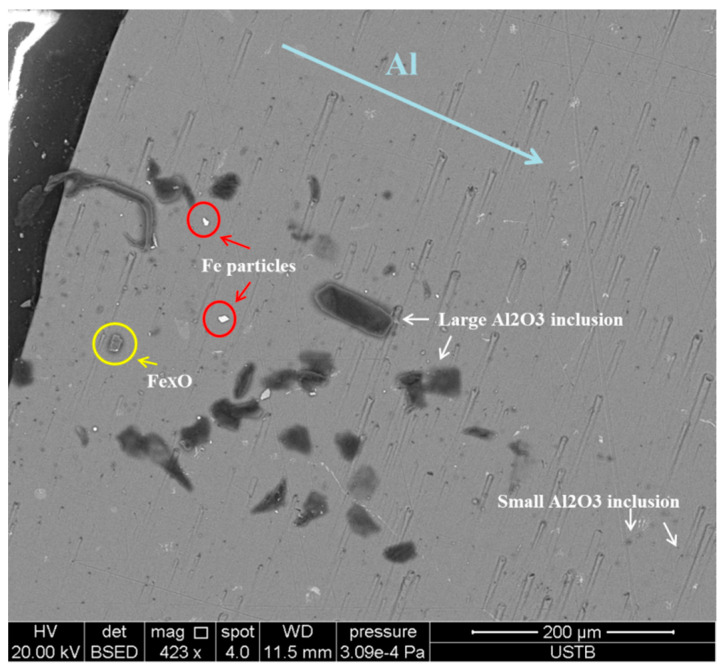
Different kinds of inclusions in Al–Fe interaction zone.

Reducing the number of inclusions in aluminum-deoxidized steel series has long been a goal of steelmaking research. With our experiments, we found that during the melting of Al alloy in molten steel, different kinds of inclusions formed in the Al matrix even if the contact time between aluminum and molten steel was quite short, as shown in Figure 7. During this process, many fine cracks formed due to the melting of the Al matrix, forming an [O] diffusion channel. Due to the strong affinity between [O] and [Al], as well as the strong diffusivity of [O], which can diffuse in the Al-rich zone to distances greater than 500 μm, [O] reacts with the melted liquid [Al] to form fine aluminum inclusions in the deep part of the Al matrix. However, in this study, the low dissolved oxygen in the aluminum matrix was the limiting step of the reaction. When the aluminum rod did not continue to melt and the oxygen diffusion channel did not change, the small-sized inclusions stopped growing.

There were also large-sized Al_2_O_3_ inclusions at the interaction surface between the Al matrix and molten steel, where the oxygen content supplied was sufficient for their formation. Small or fine Al_2_O_3_ inclusions clumped together because of the surface wettability difference between the inclusions and liquid alloy and then continued to grow. As a result, it was easier for large-sized aluminum inclusions to form. When molten steel came into contact with the Al bar, part of the former solidified, which promoted the [Fe] + [O] = (FeO) reaction occurring at the Al–steel surface. At the same time, the newly melting [Al], due to the heat transfer, reacted with the (FeO) inclusion, which also resulted in the formation of large-sized Al_2_O_3_ inclusions, in contrast to those formed following the diffusion of [Al] and [O] in the deep aluminum-rich zone of the Al matrix.

**Figure 8:**
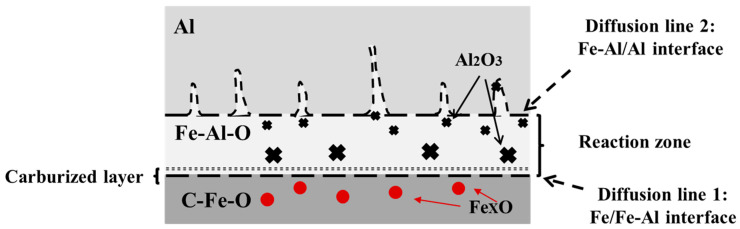
Mass transfer diagram of Al bar initial melting process.

The mass transfer diagram of the Al bar initial melting process is shown in Figure 8. The reaction zone is limited by the two dotted lines, where diffusion line 1 represents the interface between Fe and Fe-Al and diffusion line 2 that between Fe-Al and Al.

As shown at the bottom of Figure 8, Fe_x_O inclusions existed in the solid steel layer, above which a thin carburized layer formed due to the mass transfer. The line of the interface between Fe-Al and Al was not smooth and straight but had needle-like regions extending into the Al-rich zone. The local turbulence caused by different surface roughness degrees was the main reason for the formation of needle-like regions along line 2. In the reaction zone close to this line, some small-sized Al_2_O_3_ inclusions could be found, compared with the much larger-sized Al_2_O_3_ inclusions derived from [Al] and (FeO) near line 1. As can be seen at the top of Figure 8, in the Al substrate, some small, bright Fe particles could be found, and their distance from line 2 increased with the increase in immersion time.

### 3.3. Formation of Unstable AlN Inclusions in Melting Process

**Figure 9:**
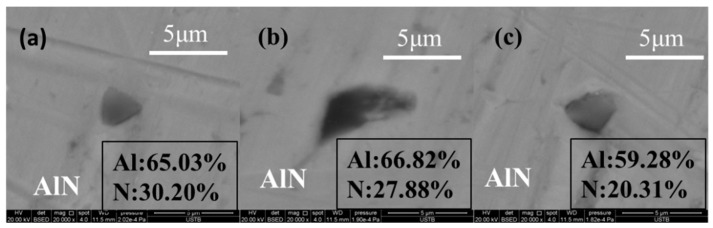
Typical AlN inclusions during melting process (**a**–**c**).

Some small-sized AlN inclusions were found in the center of the Al bar, which did not come into contact with molten steel directly during the melting process when the immersion time was 1 second. This is because the internal temperature reached the melting point of Al in the process of heat transfer, and a little amount of Al melted and reacted with the [N] in the molten steel. The size of the AlN inclusions, with some examples being shown in Figure 9, was about 5 μm.

**Figure 10:**
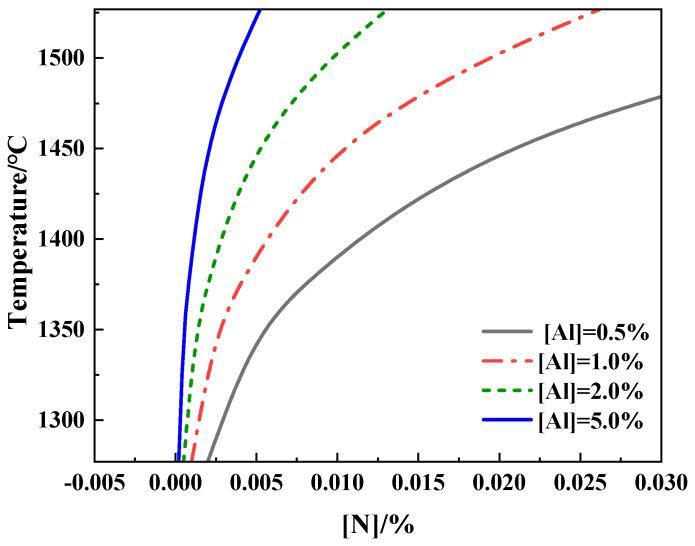
Equilibrium curves of AlN inclusion formation under different [Al] conditions.

The reaction equilibrium for the formation of AlN inclusions in liquid steel can be written as follows [22]:

(13) $${\left[\mathrm{Al}]+[N]=(\mathrm{AlN}\right)}_{s}$$

(14) $$\Delta G_{AlN}^{\Theta }=-303,500+134.6T$$

(15) $$\begin{array}{cc} \mathrm{lg}K_{\mathrm{AlN}} & =\mathrm{lg}\frac{1}{f_{\mathrm{Al}}f_{N}[\mathrm{Al}\%][N\%]}\\ & =-\mathrm{lg}f_{\mathrm{Al}}-\mathrm{lg}[\mathrm{Al}\%]-\mathrm{lg}f_{N}-\mathrm{lg}[N\%] \end{array}$$

(16) $$\mathrm{lg}f_{i}=\sum (e_{i}^{j}[j\%]+\gamma_{i}^{j}{\left[j\%\right]}^{2})$$

where *G_AlN_* is the standard Gibbs free energy of an AlN inclusion, *T* is the bath temperature (at 1600 °C), K_AlN_ is the equilibrium constant of AlN, and f_Al_ and f_N_ are the activity coefficients of Al and N, respectively. The stability of AlN inclusion formation can be calculated with the above equations. Table 2 shows the calculation process of the activity coefficients. The equilibrium curves of AlN inclusion formation under different [Al] conditions are shown in Figure 10.

Low temperatures make AlN inclusion formation easier. When [Al] is 0.5%, for AlN to form, the [N] content should be more than 0.01% at 1400 °C and about 0.005% at 1350 °C. It can also be seen in Figure 10 that higher [N] content is needed to form AlN inclusions when there is less Al content at the same temperature [24,25].

**Figure 11:**
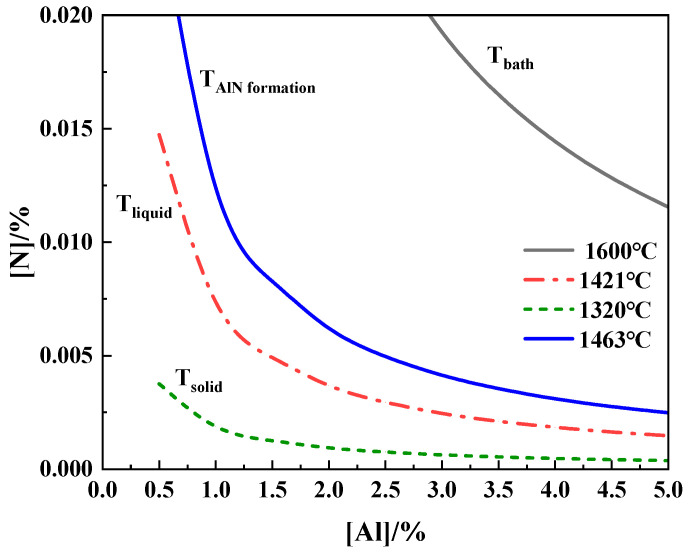
Stability diagram of AlN inclusions at experimental temperatures.

The stability diagrams of AlN inclusions at the experimental temperatures are shown in Figure 11. It can be found that there was a high probability of AlN inclusion formation in the Al-rich zone during the melting process, because the N content in molten steel was higher than that for AlN equilibrium [26,27,28,29]. However, the composition of steel was below the AlN stability line at 1600 °C, so perhaps AlN inclusions were unstable in molten steel and disappeared. T_solid_ and T_liq_ are the solid and liquid temperatures of Al, respectively, and were calculated with FactSage 7.1 software.

**Figure 12:**
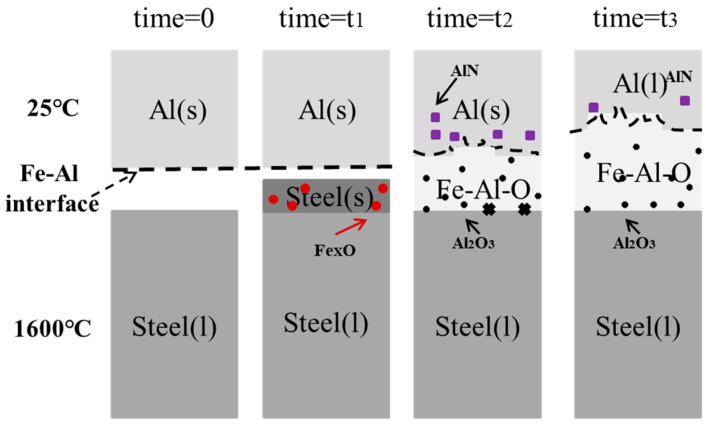
Schematic illustration of interactions between molten steel and Al bar.

Figure 12 shows the schematic illustration of the interactions between molten steel and an Al bar at t_1_, when a solidified steel layer formed around the Al bar because of the significant heat difference between the two materials. As the steel layer did not fit the interface well, a gap existed between the layer and the Al bar. Some Fe_x_O inclusions were generated in the solidified steel layer at the initial contact time, and a thin carburized layer formed at the interface between Fe and Fe-Al due to the mass transfer of [C] in molten steel. The solidified steel layer could be seen for less than 10 s because of the strong two-phase heat transfer.

Afterwards, an FeAl layer was found at the Al–Fe surface, and the diffusion layer was composed of Fe(Al)-FeAl-FeAl_2_-Fe_2_Al_5_-Al along the direction of the Al-rich matrix. Additionally, local turbulence in the molten steel caused some needle-like regions to extend into the Al-rich zone, containing dissolved [O] and [N] in contact with the Al bar; hence, Al_2_O_3_-type and AlN-type inclusions were generated in the Al-rich zone. The size of these inclusions was quite small far away from the interface due to the short Al–steel contact time. With the increase in the latter, newly melting [Al] reacted with the previously formed (Fe_x_O) inclusions, causing large-sized Al_2_O_3_ inclusions to form at the interface between Fe and Fe-Al.

At t_3_, the FeAl layer expanded with the gradual melting of the Al bar. The unstable AlN inclusions disappeared because of the constant increase in the inner temperature of the Al bar and because the [Al] and [N] contents could not meet the equilibrium condition. Finally, the Al bar melted in liquid steel.

## 4. Conclusions

The Al alloy melting behavior and interfacial reactions with steel under natural convection were investigated, and the following conclusions could be drawn based on the findings of the present study:

1. There are two routes through which the Al alloy melting process can occur. The first one has three steps: the formation of a solidified layer, the latter’s rapid melting, and Al alloy normal melting. Due to the large temperature difference and low melting point of Al, Al alloy is to be preferred for melting to occur in the inward direction. The surrounding liquid Fe cannot provide enough heat to the solid Al bar in transient time, so it turns into solid phase to release latent heat. In the second route, a solidified layer forms, the Al bar partly melts, the solidified steel layer further increases in thickness to release latent heat, and the Al bar and the solidified steel layer finally dissolve at the same time.
2. Mass transfer was investigated in this study. A thin carburized layer formed at the Fe–Al interface due to the mass transfer of carbon, which diffused from molten steel to the Al bar surface even though only 0.129% [C] was contained in the molten steel. An FeAl layer was found at the Al–Fe contact interface, and the diffusion layer was composed of Fe (Al)-FeAl-FeAl_2_-Fe_2_Al_5_-Al along the direction of the Al-rich matrix.
3. Different kinds of inclusions of various sizes could also be seen in the Al–Fe interaction area. Some Fe_x_O inclusions were generated in the solidified steel layer at the initial contact time. Afterwards, due to local turbulence, molten steel, which contained dissolved O and N, came into contact with the Al bar; hence, small-sized Al_2_O_3_-type and AlN-type inclusions were generated in the Al-rich zone. Some large-sized Al_2_O_3_ inclusions formed at the interface between Fe and Fe-Al because newly melted [Al] reacted with the previously formed (Fe_x_O) inclusions. Finally, the unstable AlN inclusions disappeared because the [Al] and [N] contents did not meet the equilibrium condition with the constant increase in the inner temperature of the Al bar.

## Figures and Tables

**Table 1:** Composition of experimental steel and Al bars (wt.%).

	C	Mn	Si	P	S	Al	Fe	Ca	Mg	O	N
Steel	0.129	1.59	0.35	0.0043	0.0030	0.018	97.7	<0.0005	<0.0005	0.0012	0.0029
Al	--	--	0.1	--	--	99.7	0.2	--	--	--	--

**Table 2:** First-order and second-order interaction parameters of components in molten steel [22,23].

	C	Si	Mn	P	S	O	N	Al
$e_{Al}^{j}$	0.091	0.0056	0.012	0.050	0.030	−6.6	−0.058	0.045
$e_{N}^{j}$	0.13	0.0470	−0.020	0.045	0.007	−0.20	0	−0.028
$\Upsilon_{Al}^{j}$	0	0	0	0	0	0	0	0
$\Upsilon_{N}^{j}$	0.014	0	0	0	0	0	0	0

## Data Availability

The original contributions presented in the study are included in the article, further inquiries can be directed to the corresponding author.
